# A penny for your thoughts: three perspectives on financial problems and their associated factors of people with psychotic disorders

**DOI:** 10.1080/17482631.2025.2479945

**Published:** 2025-03-20

**Authors:** Josephien Leonie Jansen, Vera Verhage, Richard Bruggeman, Lydia Krabbendam, Janneke Koerts

**Affiliations:** a Department of Clinical and Developmental Neuropsychology, University of Groningen, Groningen, The Netherlands; b Department of Health Sciences, Applied Health Research, University Medical Centre Groningen, Groningen, The Netherlands; c Psychosis Department, University Medical Centre Groningen, University Centre for Psychiatry, Groningen, The Netherlands; d Department of Clinical, Neuro- and Developmental Psychology, Vrije Universiteit Amsterdam, Amsterdam, The Netherlands

**Keywords:** Psychotic disorders, family members, mental healthcare professionals, therapeutic triad, financial problems

## Abstract

**Purpose:**

Financial problems are of influence on mental health, and vice versa. Indeed, finances are a key challenge for people with psychosis. To gain deeper insights into these challenges, a qualitative approach focusing on all perspectives within the therapeutic triad is needed. This study aims to investigate perspectives of people with psychosis, family members and mental healthcare professionals on people with psychosis’ financial problems, and associated factors.

**Methods:**

Fourteen people with psychosis, 15 family members and 16 professionals were recruited using purposive sampling, and participated in semi-structured, one-on-one interviews. Data was analysed using iterative thematic data-analysis.

**Results:**

Interviews revealed five themes of financial problems: Covering expenses, Financial performance, Living conditions and housing, Personal conflicts and victimization, and Regulations and legislation. Five themes were identified as factors associated with financial problems: Psychotic symptoms, Indirect factors related to psychosis, Substance use and addiction, Financial upbringing and life events, and Societal contextual factors.

**Discussion:**

People in the therapeutic triad largely mentioned similar, wide-ranging, and often co-occurring (factors associated with) financial problems of people with psychosis, risking vicious cycles. Fostering awareness and collaborative efforts among stakeholders is essential to breaking these cycles of financial problems for individuals with psychosis.

## Introduction

Challenges related to social determinants of health, including financial problems, are associated with poorer *mental* health (Kirkbride et al., 2024; Mackenbach, 2020; Thomson et al., 2022), including for people with psychotic disorders (Read, 2010; Topor et al., 2014). Reversely, psychotic disorders can have a strong negative impact on many aspects of life, such as occupational (Marwaha & Johnson, 2004), and educational performances (Rannikko et al., 2015), and social functioning (Hooley, 2010; Velthorst et al., 2017). Finance is another life domain that is a key challenge for people with psychosis (Morgan et al., 2017). As the majority of people in this group is unemployed (Hakulinen et al., 2019), they often rely on government benefits as their main source of income, resulting in considerably low annual earnings (Morgan et al., 2017). In addition, one Swiss study suggests that more than three-fourth of people with psychosis’ discretionary income is spent on buying addictive substances (Borras et al., 2007). Furthermore, gambling problems are more prevalent in this group (i.e., approximately 5.7%) compared to the general population (i.e., approximately 0.5–1.0%; Haydock et al., 2015) and are associated with an increased need for financial assistance, which might indicate financial problems. Finally, in the Netherlands, one-fifth to one-third of people with psychosis report financial dissatisfaction, which is a considerably larger proportion than in the general Dutch population (Jansen et al., 2024).

A prerequisite for financial autonomy in everyday life is having sufficient financial capability. Financial capability is defined as the “management or direction of management” of one’s finances in a way that “routinely meets one’s best interests” (Appelbaum et al., 2016, p. 5). Firstly, financial capability consists of financial competence, which is divided into financial knowledge (i.e., declarative and procedural knowledge) and financial judgement (i.e., the ability to make financial decisions that serve one’s goals). Secondly, it consists of financial performance, i.e., the ability to handle financial demands in daily life. Financial performance can be influenced both negatively and positively by contextual factors (Appelbaum et al., 2016, p. 7), such as social support, substance use, and having limited financial resources. Consequently, financial competence and financial performance may not always align (e.g., Mullainathan & Shafir, 2013; Richardson et al., 2013). Overall, financial capability is of utmost importance for independent living (e.g., for grocery shopping, saving, or handling rent) and problems with financial capability can have far-reaching consequences such as debts or poverty.

A limited number of quantitative studies directly and objectively examined financial capability in people with psychosis. Several studies assessed the subdomain of financial performance. Results suggest that people with schizophrenia show diminished financial performance compared to healthy controls (Czaja et al., 2017; Evans et al., 2003; Klapow et al., 1997; Patterson et al., 1998, 2001; Shi et al., 2013). This especially holds for people with schizophrenia with a court-appointed financial guardian (Barrett et al., 2009). Only one study directly examined financial competence in people with schizophrenia (Niekawa et al., 2007). This study suggests that people with schizophrenia score significantly lower on all domains of financial competence compared to healthy controls. However, none of these studies considered contextual factors, such as the impact of financial resources, social support or substance use. Furthermore, the studies are limited to people with schizophrenia, as opposed to the entire spectrum of psychotic disorders.

When addressing and evaluating financial problems of people with psychosis, perspectives of all important stakeholders in the therapeutic triad, i.e., people with psychosis, family members, *and* mental healthcare professionals are crucial (Cummings & Kropf, 2009; Elbogen et al., 2003, 2005; Huang et al., 2021; Webber et al., 2002). Furthermore, qualitative studies are needed to give a more detailed picture of the subject and people’s perspectives on it. Focusing on people’s narratives and unique experiences can capture not only the “what” and “how much”, but also the “why” and “how” behind their challenges (Moser & Korstjens, 2018), such as the intricacies and contextual factors surrounding financial problems. Moreover, a qualitative approach can uncover (the interplay between) financial difficulties that quantitative measures might overlook (Oranga & Matere, 2023). However, there is scarce qualitative literature addressing and comparing all these viewpoints simultaneously. What is well-documented is family burden of people caring for people with psychosis (for reviews, see Cleary et al., 2020; Dillinger & Kersun, 2020). One common aspect of family burden is financial burden (e.g., Bai et al., 2020; Caqueo-Urízar et al., 2014; Csoboth et al., 2015; Elbogen et al., 2008; Jungbauer et al., 2004; Kamil & Velligan, 2019; Lai, 2012; Lowyck et al., 2004; McCann et al., 2011; von Kardorff et al., 2016). Financial burden is caused by the direct costs (e.g., offering financial support, such as covering medical expenses), and indirect costs (e.g., loss of income due to caregiving) of family members caring for someone with psychosis (Cummings & Kropf, 2009; Elbogen et al., 2003, 2005; Huang et al., 2021). So far, only one study examining cohorts of people with schizophrenia and their caregivers in five European countries, focused directly on the perspective of family members on their relative’s financial situation, suggesting that this topic is among the most common worries among family members (Thornicroft et al., 2004).

Furthermore, mental healthcare professionals might also be involved in evaluating and supporting the personal finances of people with psychosis. For example, mental healthcare professionals are often required to evaluate patients’ financial capability and report impairments in this area (Frank & Degan, 1997; Luchins et al., 2003). In court proceedings, these evaluations can be used as admissible evidence to assign a (mandatory) representative payee (Webber et al., 2002) which, in the United States, is indeed assigned in approximately 35% of people with psychosis (Marson et al., 2006; Social Security Administration, 2022). Many professionals recognize that they should routinely consider broader financial issues (e.g., financial problems, financial burden, and financial abuse) in their contact with patients (for a review see Larkin et al., 2021) including people with psychosis (Borras et al., 2007; Woodside & Krupa, 2010). In addition, the field of financial therapy, which integrates mental health and financial counselling, is growing (Archuleta et al., 2020; Britt et al., 2015). However, the subject is still not always raised in clinical practice (Andermann, 2016; Runyan, 2018), perhaps because personal finances are a privacy sensitive subject. Other likely reasons are short consultation times or a focus on symptomatic recovery (e.g., symptom severity, side effects; Andermann, 2016; Weiner et al., 2010; Woodside & Krupa, 2010). It is, therefore, relevant to evaluate the perspectives of mental healthcare professionals on the financial problems of people with psychosis.

Finally, according to the best of our knowledge, only two qualitative studies focused on the perspectives of people with psychosis on their financial functioning. One Canadian/Australian study suggests that psychotic experiences of people with late-onset first-episode psychosis and a premorbid work history interrupted their working situation and, in turn, their financial stability (Woodside & Krupa, 2010). People in this study experienced the need to generate income as a more important motivator to return to work than symptomatic recovery. In addition, a Swedish study concludes that even though people with psychosis managed to cope with their financial challenges, these remained a continuous stressor, affecting people’s social life and sense of self (Topor et al., 2014).

In sum, financial problems are a key challenge for people with psychosis and can have serious consequences. However, there is notably scarce qualitative literature addressing and comparing the viewpoints and subjective experiences of all people within the therapeutic triad on this life domain. This joint examination of perspectives is essential, as it provides deeper insights into the unique financial challenges of people with psychosis. To fill these gaps, the current study aimed to qualitatively investigate perspectives, including both similarities and differences within these perspectives, of people with psychosis, family members and professionals, to provide a comprehensive overview of (1) people with psychosis’ financial problems and (2) factors associated with these problems.

## Method

### Participants

The present study used purposive sampling to include people with psychosis, family members, and mental healthcare professionals. People with psychosis were recruited via a mental healthcare institution and a foundation that offers occupational daytime activities to people with long-term psychiatric problems in the North of the Netherlands. People with psychosis were eligible to participate in this study when they 1) were diagnosed with a schizophrenia spectrum or other psychotic disorder (according to the Diagnostic and Statistical Manual of Mental Disorders, 5^th^ edition; DSM-5, American Psychiatric Association [APA], 2013), 2) were on a stable dose of medication prior to participation for at least four weeks, 3) were aged ≥18 years, and 4) had sufficient command of spoken Dutch to participate in the interview. In addition, they were excluded when they experienced high levels of distress due to present psychotic symptoms, and when there were indications of a severe neurological disorder (indicated in a pre-interview with potential participants or by their clinician). We aimed for a diverse sample in terms of age, sex, diagnosis, and illness duration. Data saturation (i.e., when no new, meaningful data emerged from the interviews) was reached when 14 people with psychosis (with a mean age of 41.1 years, SD = 13.9, range = 24–64, and a mean illness duration of 13.1 years, SD = 13.3, range = 1–39) were included (Table I).

**Table I.** Participant details of people with psychosis, family members and mental healthcare professionals.

People with psychosis	Gender	Diagnosis	Primary working situation	Net monthly income^a^
P01	Male	Schizophrenia	Student	€1000 - €2000
P02	Male	Delusional disorder	Work experience placement	€1000 - €2000
P03	Male	Psychotic disorder NOS	Labor-based daytime activities	€1000 - €2000
P04	Female	Schizophrenia	Labor-based daytime activities	< €1000
P05	Male	Schizoaffective disorder	Labor-based daytime activities	€1000 - €2000
P06	Male	Schizophrenia	Labor-based daytime activities	€1000 - €2000
P07	Male	Schizophrenia	Labor-based daytime activities	€1000 - €2000
P08	Male	Delusional disorder	Unemployed	€1000 - €2000
P09	Female	Brief psychotic disorder	Disabled to work	< €1000
P10	Female	Brief psychotic disorder	Employed	> €2000
P11	Male	Schizophrenia	Labor-based daytime activities	€1000 - €2000
P12	Male	Schizoaffective disorder	Labor-based daytime activities	€1000 - €2000
P13	Male	Psychotic disorder NOS	Employed	< €1000
P14	Male	Psychotic disorder NOS	Employed	€1000 - €2000
Family members	Gender	Diagnosis relative	Gender relative	
F01^b^	Female	Schizophrenia, schizoaffective disorder	Female, Male	
F02	Female	Drug-induced psychotic disorder	Male	
F03^b^	Female	Schizophrenia, schizophrenia	Male, Male	
F04	Female	Schizophrenia	Male	
F05*	Male	Schizophrenia	Male	
F06*	Female	Schizophrenia	Male	
F07	Female	Psychotic disorder NOS	Male	
F08	Female	Schizophrenia	Male	
F09	Female	Schizophrenia	Male	
F10	Female	Schizophrenia	Female	
F11	Female	Schizoaffective disorder	Male	
F12	Male	Psychotic disorder NOS	Male	
F13	Male	Delusional disorder	Male	
F14	Female	Bipolar disorder	Male	
F15	Male	Delusional disorder	Male	
Healthcare Professional	Gender	Profession	Work experience	
MHP01	Male	Psychiatrist	20 years	
MHP02	Male	Mental health nurse	41 years	
MHP03	Female	Psychologist	2 years	
MHP04	Female	Social worker	6 years	
MHP05	Male	Mental health nurse	10 years	
MHP06	Female	Mental health nurse	26 years	
MHP07	Male	Job coach/counsellor	8 years	
MHP08	Female	Job coach/counsellor	10 years	
MHP09	Female	Psychiatrist	2 years	
MHP10	Male	Medical doctor	2 years	
MHP11	Female	Nurse practitioner	24 years	
MHP12	Male	Psychiatrist	27 years	
MHP13	Female	Psychologist	7 years	
MHP14	Male	Mental health nurse	16 years	
MHP15	Female	Nurse practitioner	Non-disclosed	
MHP16	Female	Mental health nurse	10 years	

*p* = Person with psychosis; NOS = Not Otherwise Specified. F = Family member, MHP = Mental Healthcare Professional.

^a^The net modal monthly income in the Netherlands at the time of data collection was approximately €2,300 (Statistics Netherlands, 2024). ^b^Participant has multiple family members with psychosis.

*Participants are a couple.

Family members were recruited via a mental healthcare institution in the North of the Netherlands, and a national family member association of people with psychosis. They were included when they 1) were a family member of someone with a diagnosis in the schizophrenia spectrum (APA, 2013); 2) were aged ≥18 years, 3) had sufficient command of spoken Dutch to participate in the interview, and 4) had no (self-reported) diagnosis in the schizophrenia spectrum (APA, 2013). They were not required to be family members of the people with psychosis participating in this study. We aimed for a diverse sample in terms of illness duration of, and relationship to the person with a psychotic disorder. Data saturation was reached when 15 family members (with a mean age of 67.5 years. SD = 9.4, range = 44–82) were interviewed (Table I). The mean illness duration of their relative with a psychotic disorder was 18.4 years, SD = 12.8, range = 2–42. Among these 15 family members were 10 mothers, 3 fathers, 1 brother, and 1 sister-in-law.

Lastly, mental healthcare professionals of people with psychosis were recruited via several mental healthcare institutions in the North of the Netherlands. We aimed for a diverse sample in terms of age, sex, type of healthcare profession and work experience. Data saturation was achieved when 16 professionals (with a mean age of 43.7 years, SD = 9.4, range = 32–65) were interviewed (Table I).

### Material

#### Interview guide

An interview guide was developed prior to the interviews based on scientific literature on financial capability (e.g., Appelbaum et al., 2016; Niekawa et al., 2007) and the researchers’ experience with this topic. In addition, an advisory group of experts by experience, family members and professionals was formed who gave suggestions for the guide’s improvement. For example, they suggested giving concrete examples when discussing abstract topics (e.g., *How do you handle large expenses, such as going on holiday?)*. The types of questions asked included e.g., *Have you ever experienced financial problems? Can you give an example? What caused these financial problems to arise?* While the semi-structured approach guided the interview, it was aimed to give the researcher flexibility to probe responses, giving participants the opportunity to tell their unique story (Smith, 1995).

#### Demographic characteristics

All participants were asked to state their age (in years) and sex (male, female, or other). People with psychosis were asked to state their diagnosis in the schizophrenia spectrum, illness duration (in years), primary working situation and net monthly income (six discrete categories: below €1000, €1000–€2000, €2000–€3000, €3000–€4000, €4000–€5000, and above €5000). Family members were asked to state their relationship to the person with psychosis and their relatives’ gender (male, female, or other), diagnosis in the schizophrenia spectrum, and illness duration (in years). Professionals were asked to state their profession and work experience (in years).

### Procedure

People with psychosis could contact the first author if they were interested in participating. Alternatively, mental healthcare professionals asked eligible people with psychosis for (verbal) permission to be contacted by the first author. They received an information letter, and had an informative pre-interview by phone. Family members and professionals contacted the first author themselves and also received an information letter. In the pre-interviews, participants were informed about the study, inclusion criteria were checked, and participants had the opportunity to ask questions. An interview was scheduled after a reflection period of at least two weeks. After participants provided their written informed consent, in-depth, face-to-face interviews were conducted. Each interview was scheduled for 45–90 minutes and was held at a location chosen by the participant. To establish rapport, all interviews began with an introduction and explanation of the project. Participants were ensured that their identities would be confidential and that data would be de-identified. First, demographic questions were asked. The interviews were audio recorded and afterwards, the recording was transcribed verbatim. A narrative summary report of the interview was returned to participants for review (i.e., member check). The local Central Ethical Committee approved the study (Research no. 202100079).

### Data-analysis

The Qualitative Analysis Guide of Leuven (Dierckx de Casterlé et al., 2012) was used as a guide for qualitative data-analysis. Underlying principles include 1) a case-oriented approach, in which within-case and cross-case analysis are continuously balanced (e.g., Ayres et al., 2003), and 2) a constant-comparative approach; the iterative process of verification of the analysis (i.e., developing ideas, codes, and themes) against the data (e.g., Frogatt, 2001). The analysis followed two phases and six overall steps (Braun & Clarke, 2006).

In the preparatory coding (i.e., case-oriented, more narrative) phase, the first and last author read, re-read and annotated each transcript several times to become familiar with the text and to identify preliminary codes (step 1). Each transcript and the narrative summary reports were discussed with the research team and based on these discussions, a conceptual interview scheme was created, in which narrative descriptions were formed into more abstract, conceptual concepts (step 2). Each transcript was reread with these interview schemes in mind to refine these schemes. As a final step in the preparatory phase, the concepts from the interview schemes were refined and developed through comparisons within and across interview schemes (step 3).

The actual coding phase follows the process of thematic analysis, in which the interviews were coded line-by-line using Atlas.ti 23.2 (step 4). Coding was data-driven (inductive). To ensure rigour and provide methodological oversight, an independent researcher, (the second author), who was not involved in the initial study design or data-collection, contributed to this process. All concepts were described and integrated into themes (step 5). These themes were constructed iteratively, moving back and forth between de codes and the original transcripts, and through thorough discussions with the research team, to ensure an accurate representation of participant’s experiences. Lastly, themes were refined after peer debriefing. Each theme was given a descriptive name and illustrated with relevant participant quotations (step 6).

To ensure good quality of our analysis, we used the quality criteria credibility, dependability, confirmability, and transferability (Creswell & Poth, 2016; Lincoln & Guba, 1985). Member checking achieved credibility of the data. Participants had the opportunity to check the compatibility of the results with their experiences. Nine out of 14 people with psychosis, 12 out of 15 family members and 11 out of 16 professionals used this opportunity. Mostly, participants had no or minor comments. The first and last author evaluated all comments. We achieved dependability through external checks on concepts and codes by the second author, whereby a sample of the interviews were independently coded. Regular meetings were held to discuss coding decisions and reach consensus. In addition, we achieved dependability through careful and transparent documentation of the process of data collection and data analysis throughout the study. Confirmability was achieved by logging the process from raw data, via data reduction and combination to data reconstruction in Atlas.ti. Lastly, transferability was achieved through a thick description of participant’s selection, demographic data, data collection, results, and context.

### Reflexivity

To reflect on the underlying assumptions about finances and financial problems, the first author wrote memos throughout the data-collection phase. One recurring topic in the memos was the author’s privileged position as a highly educated woman who had a stable financial upbringing and current financial security. This privileged background can result in prejudice about this topic (‘S Jongers, 2024). Therefore, the first and last author engaged in regular discussions about the possible impact of this perspective, with the goal of fostering empathy during the interviews. In addition, prior to each interview, the first author employed in bracketing techniques (Tufford & Newman, 2012), noting her initial thoughts about the interview and/or the interviewee. This practice enabled her to attend each interview as open-minded as possible.

## Results

**Figure 1.**
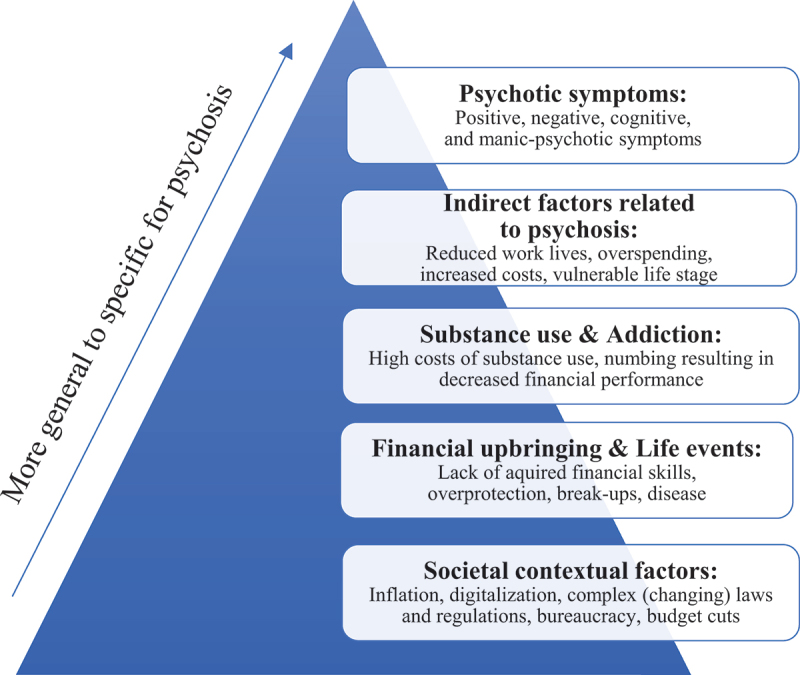
Visual representation of the factors associated with financial problems of people with psychosis according to people in the therapeutic triad.

Themes were formulated regarding research question 1: financial problems of people with psychosis, and research question 2: factors associated with these problems. Five themes regarding financial problems emerged from the interviews. In addition, five themes were generated as factors associated with financial problems, ranging from factors closely related to psychosis to more general factors (Figure 1).

### Research question 1: financial problems of people with psychosis

It is important to note that participants in all groups often mentioned multiple, interrelated financial problems that interacted with and amplified each other’s impact. “*It actually turned into a sort of mishmash of things piling up. Finances piling up, and problems piling up.”* (P03). On the other hand, participants highlighted the absence of financial problems at least at some point in the person with psychosis’ lives. Mostly, this was due to the (temporary) lack of associating factors or the presence of protective factors (e.g., social network offering assistance).

#### Covering expenses

Problems with covering expenses is a key issue for people with psychosis, highlighted by participants in all groups. This is a problem in an *absolute* sense, where people lack the resources to purchase necessary items (e.g., food, toiletries). *“I once stole a [personal hygiene essential] because I didn’t have any left.”* (P04). Participants also expressed limited resources in a *relative* sense, when compared to people from the same surroundings. For example, people were unable to engage in certain social activities, and described feelings of shame and feeling unable to fully participate in society or keep up with their peers. *“Accepting it. Yes, that’s definitely a good one. But I find it really difficult because … (…). I just want so badly to do what other people do too.”* (P09). In some cases, it negatively affected people’s treatment. *“I occasionally provide a specific treatment, and in some cases, they are required to purchase a book, for instance, and some individuals simply cannot afford it.”* (MHP03).

Additionally, participants in all groups consistently mentioned that people with psychosis can end up in the red or accumulate substantial debts. Once in this situation, it can become exceedingly difficult to escape it, which can result in a high burden and feelings of distress, stigma, and shame. “*Just piling up, piling up, bailiff, bailiff, higher costs, another bailiff, even higher costs.”* (F03). The feelings of financial distress related to limited spending power or debts could, in turn, influence financial performance (see below). As P06 described: *“ … Bills were left unpaid. And I didn’t open my mail either, so my mailbox was [full], and I would empty it into a trash bag and store it under my bed. (…). To avoid being confronted with payment reminders and such”*.

#### Financial performance

Participants with psychosis, family members, and professionals stressed problems with financial performance as another key problem of people with psychosis. Specific problems with financial performance mentioned by participants in all groups include lacking financial oversight, not paying bills/rent or paying bills/rent late, not opening mail, and short-term thinking: *“Within two years, that money was completely spent, when he was in the clinic. (…). He couldn’t say ‘well, now I can use this for the next ten years… and I won’t have financial problems anymore’.”* (F01). Family members and professionals mentioned that people with psychosis refrained from performing certain financial actions. For example, some people refrained from applying for unemployment benefits when they lost their jobs, or from terminating their (student) loans or tuition fees, despite having a psychotic episode that prevented them from working or studying: “*For example, even with their studies, not attending for whatever reason, but still borrowing [student loans] heavily. They don’t have the idea that, ‘Hey, this has to be paid back’.”* (MHP05).

Importantly, in addition to problems with covering expenses influencing financial performance, participants in all groups mentioned the reverse: a lack of financial oversight could lead to problems with covering expenses, mainly because it led to someone spending more money than they earned.

In addition to these examples of specific problems with financial performance, participants consistently described more general problems with financial performance. They provided statements such as people “struggle to manage money” without being able to give details. Participants, furthermore, described that as a consequence, people with psychosis might require (mandatory) financial assistance from a family member or a representative payee: “*I think they might not have the cognitive ability for it, so they don’t plan and… But then they receive weekly allowances. Some get daily allowances. So, that’s how it’s compensated for.”* (MHP13).

#### Living conditions and housing

Both people with psychosis and professionals reported reduction in self-care, deferred maintenance, and household pollution, highlighting the interplay between financial issues and overall living conditions of individuals with psychosis. MHP14 stated: “*This lady, living in a concrete space in her apartment, has nothing on the floor (…) she doesn’t have much money, and then we requested [i] if she could get a washing machine and carpet (…).”* Family members described reduction in self-care, but they did not mention deferred maintenance or household pollution.

Participants, furthermore, reported homelessness: *“I was homeless for three years, yeah, I spent all my money to buy drugs and alcohol.”* (P12). Also, (the risk of) house eviction (e.g., due to rent arrears) of people with psychosis was mentioned: “*And then my mother also provided financial assistance because otherwise, he wouldn’t have been able to pay his rent, and it wouldn’t have been long before he would have been evicted from his house.”* (F13). Some family members specifically described how their relatives started roaming the streets without informing their family, for example during a psychotic episode.

#### Personal conflicts and victimization

Particularly family members and professionals described personal conflicts about finances. In most cases, these conflicts arose between the individual with psychosis and a family member, when the role of caregiver and/or representative payee interfered with their personal relationship or parenting role. For family members, this was often experienced as a high burden with mental and physical consequences: *“He kept coming back to me for money, and ultimately, (…) I said I won’t do it [help financially] anymore. You can end up on the streets, I don’t care anymore. (…) Yes, but I had to do it [cut off financial help], otherwise I would have been ruined myself.”* (F07). Due to this burden, family members sometimes received or would like to receive (mental) healthcare: *“I think that parents also need emotional support when… it’s already so difficult to deal with your psychotic family member in daily life. And I also have to deal with those finances, there should be more support for that.”* (F10). In contrast, people with psychosis hardly described certain conflicts, seldom mentioning the burden on their relatives.

In addition to these conflicts, participants in all groups noted that people with psychosis can be victimized, or that they are particularly vulnerable to victimization in a financial context. *“The owner had a van at the side of the road, and he wanted to sell it to me. He wanted to transfer it to my name as soon as possible. I just didn’t notice. (…). He was a scammer.”* (P11). Examples of financial victimization are being victim of swindles, exploitation, or financial mismanagement by a family member or (mandatory) representative payee: “*The representative payee didn’t advocate well for my child, left old bills unpaid while paying new ones, which resulted in him accumulating high, very high debts.”* (F02). In some cases, participants indicated that financial victimization resulted in increased emotional and psychological distress, including increased psychotic experiences.

#### Regulations and legislation

The problems associated with this theme were described by people with psychosis and family members, not by professionals. According to these participants, certain (government) regulations can result in high bills and therefore contribute to financial difficulties. Specifically, participants mentioned repaying security deposits or government subsidies: *“I still have my children living with me, and my son earns enough that I had to repay my rent subsidy last year. So, I had a lot of money in the bank (…), but it actually all went back there.”* (P09).

Furthermore, people with psychosis and family members described problems with legislation, such as law violations with a financial motive (e.g., to acquire money when having debts). Law violations that were mentioned were theft, burglary, and drug trafficking. Although it appears to be rare, some participants mentioned that people with psychosis are being fined or even sentenced to prison or a custodial mental institution because of these violations. F04 said: *“At one point, he got arrested and sent to prison. And yeah, because he had also done certain things, including assaulting people (…), he had to pay damages because he had assaulted someone.”*

### Research question 2: factors associated with financial problems of people with psychosis

Similar to the financial problems described above, participants in all groups mentioned various factors associated with financial problems of people with psychosis that can interact with and amplify each other. For example, life events might trigger psychotic symptoms or substance use, while substance use could in turn exacerbate psychotic symptoms. The factors ranged from factors specific for people with psychosis to more general factors (see Figure 1). In this context, participants offered nuanced insights, noting that financial problems can exist independently of a psychiatric diagnosis, and are often shaped by individual differences and societal factors. Notably, participants in all groups mentioned the distress associated with financial difficulties negatively impacted mental health, including suffering that participants considered as psychotic symptoms: *“When people genuinely worry about their finances, it can, of course, be a reason for them to become more mentally unstable in terms of psychosis.”* (MHP09).

#### Psychotic symptoms

Professionals, people with psychosis, and family members consistently mentioned positive symptoms being associated with financial problems. Especially, disorganization is a factor that is related to a lack of financial oversight. In addition, participants noted financial problems due to hallucinations, such as hearing voices that tell people to spend money. More regularly, people got into financial problems due to a delusion, such as grandiose delusions or paranoia: *“He started filling out my mother-in-law’s tax forms because he believed he was a tax advisor and, by that time, he considered himself a lawyer, which he wasn’t. (…). So, after a few years, my mother-in-law suddenly received a tax bill stating that she had to pay more taxes, and her housing subsidy was revoked (…).”* (F03). Or MHP10 stated: *“I remember one case (…) of someone who constantly felt like they had to go to [island] to escape 5 G. Well, every time, the ferry and the taxi ride. We noticed that it was really bothering his mother that the finances had skyrocketed, and she couldn’t handle it.”*

Participants in all groups also noted behaviours that can be identified as negative symptoms, particularly avolition, as a factor associated with financial problems, specifically problems with financial performance: *“(…) You just get this reminder that you have to pay it now, but I was really like, okay, I want to pay, but I just couldn’t do it. I’m not quite sure how to put it into words, but, well, it just wouldn’t happen.”* (P10). Additionally, family members and professionals, but no participants with psychosis, described cognitive problems (e.g., problems with concentration) as being associated with financial problems: “*There’s also cognitive decline. So (…) that’s one of the things that eventually doesn’t work well anymore. You know, how you handle money.”* (MHP04). While cognitive decline is commonly associated with psychosis, it was mentioned that the use of (antipsychotic) medication may also be associated with these cognitive problems. Lastly, family members and professionals reported manic-psychotic episodes as being associated with financial problems. Manic-psychotic episodes were particularly mentioned related to problems with covering expenses, due to overspending: *“Well, when someone is manic-psychotic, they might think that everything will be fine, that money should be spent freely, and they start spending money that isn’t theirs.”* (MHP14).

#### Indirect factors related to psychosis

People’s psychotic symptoms were also indirectly related to financial problems. Participants consistently described that psychosis influences people’s working lives. Often, people lose their jobs and have to rely on government benefits. Alternatively, participants noted that people might work less or on a lower level due to psychotic symptoms. This might result in people with psychosis experiencing a sense of loss, or inequality with their peers. P05 stated: “*It can sometimes overwhelm me, of course, when I think about it. I have a [high school] diploma, I attended a university of applied sciences, which I didn’t finish (…). If I had passed everything and hadn’t gotten sick, I might have had a well-paying job. People I know from my class or fellow students, they’re all doing very well. And when I compare myself to them, I think, yeah, I could have had that too.”* In case people return to work after a period of absence, their income might not (significantly) improve due to a loss of benefits: *“Well, that lady is now hesitant to work because she had a bad experience in the past where she ended up worse off financially by working.”* (MHP08).

In addition, participants in all groups consistently mentioned overspending as being indirectly related to symptoms of psychosis. For example, family members and professionals specifically mentioned that people overspend as a coping mechanism, i.e., they spend money to lessen the effect of their symptoms, improve their self-esteem, or as a way to contribute to society. F05 said: “*If you have a life where it’s hard to get a grip on reality, where things you undertake fail, and you constantly receive negative feedback, then it is, of course, wonderful if you occasionally get some positive feedback. When do you get that? When you are approached on the street by someone who wants to make you a subscriber to something. And then (…) you naturally receive a lot of positive feedback because you participate.”*

Another indirect factor related to psychosis mentioned by participants in all groups is increased costs related to having psychosis. Participants particularly emphasized increased and/or unexpected healthcare costs: *“I had my healthcare deductible set at 800 euros. (…). And then I ended up here [at the hospital], and I still have to repay that (…).”* (P01).

Lastly, a point raised particularly by professionals is that a psychotic disorder often emerges during early adulthood, a stage in life during which people are especially vulnerable to also getting financial difficulties: *“The adolescence and young adulthood phases are very crucial periods in your personal development, so when things start going a little awry during that time, the chances of it going even more awry naturally increase. What I notice with the somewhat younger group is that they really want to be ‘normal’. They want to have a car, regular employment, go out on weekends, go on vacations, and have more financial freedom.”* (MHP11).

#### Substance use and addiction

Participants in all groups consistently mentioned substance use and addiction being associated with financial problems. Most problems with substance use and addiction were due to the use/abuse of cannabis or alcohol. The use of tobacco and hard drugs and gambling were also noted by participants. The main reason for substance use and addiction to lead to financial problems, is the high costs: *“I have to say, when it came to that addiction, it was a bit of a slippery slope (…), like a snowball that kept getting bigger in terms of expenses.”* (P02).

Furthermore, substance use can be associated with problems with financial performance, as people are less able to deal with financial matters when they use substances. MHP07 said: *“If someone (…) realizes that something is not right with them, that’s often the trigger to start using marijuana, to numb themselves. And then (…) end up in such a daze that opening the mail is no longer a priority.”*

#### Financial upbringing and life events

With regard to financial upbringing, participants consistently described a lack of acquired financial skills from parents as an associated factor. P12 stated: *“I only had a mother. She didn’t pass anything down to me from home. Nothing, just food and drink.”* Factors associated with the lack of acquired financial skills in people’s upbringing include traumatic events, being raised by a single parent, or having (a) parent(s) with mental health problems. Alternatively, when family members offer too much financial assistance, such as giving the person with a psychotic disorder money while it is not spent responsibly, this “overprotection” might also be associated with financial problems according to professionals: *“Yeah, you know, then I’m being confrontational. ‘Oh, so your father is financing your weed?’ (…) That’s almost neglect; just constantly giving someone money and yeah, that father… I can’t imagine he doesn’t have some idea of what’s going on with it. But yeah, I think, setting boundaries. Saying once: no, you’re not getting any money. That shows more involvement than just transferring €100 every month.” (MHP06)*. Lastly, participants in all groups described life events, such as a divorce, break-up, or a severe disease, as factors that, especially when co-occurring with a psychotic episode or vulnerability, are associated with financial problems. P09 said: *“When I got divorced, I returned to living from social welfare, and that’s where I’m still at, actually. And up until last year, I thought, ‘Now, I want to get out of this, I want to find a job, and I want to make something of my life.’ Yeah, and then the psychosis came into my life.”*

#### Societal contextual factors

Only family members and professionals noted societal factors that are associated with financial problems of people with psychosis. Inflation was mentioned as one societal factor that might increase financial difficulties. In addition, professionals mentioned increasing digitalization as a challenge, especially for older people with psychosis. Lastly, professionals emphasized that the complex, frequently changing laws and regulations (e.g., regarding benefits, social security) and bureaucracy in the Netherlands can make it difficult for people to manage their finances well: *“It’s the bureaucracy that makes you have to go there in person and fill out a form just to block your debit card; that’s all quite cumbersome, especially when you’re confused. What a hassle.” (MHP16)*.

Once financial problems occur, several participants pointed to broader societal developments that negatively impact subsequent effective financial assistance and support. For example, budget cuts in the healthcare sector were mentioned, leading to reduced availability of social workers.

## Discussion

Financial problems are a key challenge for people with psychosis, but only a few studies on this topic exist. Especially, the combined subjective experiences from people in the therapeutic triad related to financial problems hardly received attention in scientific literature. This study is, therefore, the first to qualitatively investigate and compare the perspectives of people with psychosis, family members and mental healthcare professionals on people with psychosis’ financial problems. Additionally, factors associated with these problems were examined. Interestingly, our results suggest that participants across all groups generally identified comparable financial problems for people with psychosis including problems with covering expenses, financial performance, living conditions and housing, and personal conflicts and victimization. People with psychosis and family members, but not professionals, also noted problems with regulations and legislation. These similarities could suggest that all members of the therapeutic triad generally have reasonably good insight into the financial difficulties people with psychosis face and are able to reflect on their own role or shortcomings in this regard. For example, people with psychosis reflect on their financial performance, and family members recognize their role in conflicts about finances. The similarities in the identified themes, combined with insight and reflection of people within the therapeutic triad is valuable if they are to collaborate to prevent or address financial problems. It must, however, be noted that people with psychosis barely mentioned the burden financial conflicts put on their family members. Greater acknowledgement of this burden could help alleviate it. In addition, professionals did not mention problems with regulations and legislation, while people with psychosis and family members did identify issues in this domain. Thus, professionals are encouraged to acknowledge potential legal issues (related to finances) and their potential consequences within the context of mental healthcare.

Notably, while most of the problems identified here are not necessarily specific for people with psychosis, this study gives a comprehensive overview of co-occurring, and often serious financial problems which amplify each other’s impact. For example, limited financial resources (i.e., problems with covering expenses) can reduce one’s mental bandwidth, which might result in problems with financial performance (e.g., not opening mail, short-term thinking; Mullainathan and Shafir (2013)), which in turn might increase problems with covering expenses in the long run, or even the risk of homelessness. This indicates the risk of vicious cycles related to financial problems. Thus, preventing and addressing these (cycles of) financial problems is crucial, as our results indicate that they can have substantial consequences, such as for societal participation, leisure activities, mental health, and (access to) healthcare.

The financial problems identified in the current study partly align with various problems described–separately-in previous research in people with psychosis. Problems with covering expenses are consistent with studies showing that people with psychosis have low annual earnings (Morgan et al., 2017). Financial performance problems are relatively well-documented in the subgroup of people with schizophrenia (Barrett et al., 2009; Czaja et al., 2017; Evans et al., 2003; Klapow et al., 1997; Patterson et al., 1998, 2001; Shi et al., 2013). Additionally, in the United States, approximately 35% of people with psychosis require assistance from a (mandatory) representative payee (Marson et al., 2006; Social Security Administration, 2022). Furthermore, (being at risk of) homelessness in this group is well-documented (Felix et al., 2008; Foster et al., 2012; Lin et al., 2022; Odell & Commander, 2000; Ran et al., 2006), with no income as an important risk factor (Ran et al., 2006). Also, limited research suggests personal conflicts and aggression due to financial dependency on a caregiver (Elbogen et al., 2005, 2008). Lastly, extensive research indicates problems with regulation and legislation, reporting an increased risk of law violations of people with psychosis compared to the general population (Whiting et al., 2022; Yee et al., 2020). These include crimes to ensure people’s basic survival (Martinez, 2023), for which low socio-economic status, low income and unemployment are important risk factors (Lamsma & Harte, 2015). Financial problems identified in the current study, but less-explored in previous literature include problems with living conditions (e.g., reduced self-care, household pollution) and financial victimization (e.g., scams, fraud). Dutch studies suggest that people with psychosis are victimized approximately four to six times more often than people in the general population (de Vries, Pijnenborg, et al., 2019; de Vries, van Busschbach, et al., 2019). However, these studies primarily focused on violent or sexual crimes, often overlooking financial victimization. Thus, our results align with, but also complement previous research. When important stakeholders are more aware of the co-occurring and compounding financial problems of people with psychosis, they can recognize these earlier, which can help breaking the vicious cycle.

Overall, people with psychosis, family members and professionals also mentioned similar factors that are associated with the financial problems described above. These factors range from factors specific for psychosis to more general factors: psychotic symptoms, indirect factors related to psychosis, substance use and addiction, and financial upbringing and life events. Family members and professionals, but not people with psychosis, also identified societal contextual factors (e.g., inflation, bureaucracy) as being associated with financial problems. Again, the similarities could indicate reasonably good insight and ability to reflect on this topic in people in the therapeutic triad e.g., where people with psychosis reflect on the high expenditure on substances, and professionals recognize the problems associated with high healthcare costs. On the other hand, it could be helpful for people with psychosis to be made aware of the potential influence of more general, societal contextual factors on their financial problems. Such awareness can potentially mitigate self-blame and self-stigma, allowing people to attribute less blame and responsibility to themselves for their financial difficulties. Additionally, it must be noted that although participants generally mentioned similar (factors associated with) financial problems of people with psychosis, these are not experienced similarly. People with psychosis face daily struggles and distress associated with financial problems. Yet, some expressed relative financial satisfaction despite having, for example, a low income. One reason for this is that, partly due to financial support, they experienced more freedom and less financial stress. In contrast, family members frequently expressed negative emotions in the interviews, highlighting the substantial burden that (offering support with) their loved one’s financial challenges has placed on them. This burden goes mostly unnoticed by people with psychosis in the interviews. On the other hand, professionals observed these challenges from a distance. Despite acknowledging the importance of recognizing financial problems, professionals in the interviews noted that these issues are not *routinely* discussed in clinical practice. They also expressed uncertainty about whether prioritizing financial problems falls within the scope of mental healthcare responsibilities. Studies suggest that short consultation times and a focus on symptomatic recovery contribute to these considerations (Andermann, 2016; Weiner et al., 2010; Woodside & Krupa, 2010).

Reflecting on the identified associated factors, manic-psychotic episodes are unsurprising, as excessive buying is a diagnostic criterion in schizo-affective disorders (DSM-5; APA, 2013). In addition, indirect factors related to psychosis, such as unemployment (Hakulinen et al., 2019; Woodside & Krupa, 2010) and high healthcare costs (Csoboth et al., 2015; Huang et al., 2021; Yu et al., 2017) are well-recognized in previous studies. In the current study, substance use, mainly cannabis and alcohol, is mentioned consistently by participants in all groups as being associated with various financial problems. Previous studies on people with psychosis have also recognized substance use and addiction as an associated factor (Borras et al., 2007; Desai & Potenza, 2009; Haydock et al., 2015; Jansen et al., 2024; Machart et al., 2020). However, many of these studies centred on gambling addiction. In contrast to these studies, gambling problems received far less emphasis compared to the influence of substance use on financial difficulties in the present study. The other factors highlighted in this study (e.g., financial upbringing, life events, and societal contextual factors), including factors unique for people with psychosis (i.e., psychotic symptoms), received surprisingly little attention in the scientific literature.

Indeed, psychiatric symptoms are mostly recognized as being indirectly related to financial problems (e.g., Lees & Stacey, 2024). For example, the following categories of (often co-occurring) causes for financial problems are described in general population (Jungmann & Madern, 2021): (1) survival (i.e., when fixed expenses invariably exceed income), (2) compensation (i.e., coping with stressful situations by abundant spending), (3) adaptation (i.e., a major change in income due to life events), (4) overspending (i.e., living beyond one’s means), and (5) bureaucracy (i.e., bureaucratic inability to manage finances). These categories party overlap with the current findings, but the authors describe psychiatric problems only as an indirect stressor leading to abundant spending (i.e., compensation), or a reason for income decline (i.e., adaptation). The current study, however, suggests that specifically psychotic symptoms are also directly related to financial problems, e.g., people’s positive, negative, manic-psychotic or cognitive symptoms can result in problems with covering expenses or financial performance. Thus, although also not all associated factors are exclusive to people with psychosis (Figure 1), several factors, including psychotic symptoms, can heighten their susceptibility to financial problems.

Conversely, disadvantageous social circumstances, including financial stressors, can substantially impact people whether they live with a diagnosed mental illness or not (Mood and Jonsson, 2016). Financial problems are also closely related to (the exacerbation of) mental health problems (Kirkbride et al., 2024; Topor et al., 2014; Priebe, 2016), particularly among those living on marginalized circumstances, such as individuals with the lowest incomes (Bond & D’Arcy, 2021; Kirkbride et al., 2024; Thomson et al., 2022). Our results also suggest that financial stressors may elevate suffering, which, by our participants, was interpreted as psychotic symptoms. Taken together, this bidirectional association between personal finances and mental health highlights the potential for another vicious cycle, and calls for caution to avoid unnecessarily pathologizing behaviours that stem from broad socioeconomic distress and vulnerabilities (Topor et al., 2014, 2019; Johnstone & Boyle, 2018).

Beyond examining the perspectives of people with psychosis, family members, and mental healthcare professionals, in this regard, it is crucial to critically reflect on our perspective as researchers with a background in (mental) healthcare throughout the research process. Peer debriefing alerted us to a *psychiatric bias*, i.e., a tendency to over-attribute maladaptive financial behaviours (e.g., not opening mail) to psychotic symptoms (e.g., negative symptoms), and undervaluing the reverse relationship—the impact of financial problems on behaviour and mental health. These findings and feedback underline the importance of critical awareness of all perspectives and the use of a holistic approach, in contrast to a more reductionist approach that focuses primarily on the symptoms of psychosis and its consequences. Furthermore, they underscore the need to recognize the complex interplay of wide-ranging factors associated with financial issues, including factors specific for psychosis, while also timely addressing and detecting the financial stressors in this group to prevent worsening of both financial problems and psychiatric symptoms.

This holistic understanding is well-supported by the model of financial capability (Appelbaum et al., 2016). Participants in the current study described financial problems and associated factors on every level of the model. Financial performance is one of the key challenges mentioned by our participants. Additionally, participants mentioned themes related to financial competence, such as a lack of acquired skills in people’s upbringing (i.e., financial knowledge), and spending money on substance use (i.e., financial judgement). However, most themes are associated with contextual factors (e.g., homelessness, regulations, social conflicts and victimization, life events and societal contextual factors). Thus, professionals are advised to adopt a holistic approach to evaluate financial problems in people with psychosis. This includes assessing people’s financial performance and competence, but also identifying influential contextual factors, including factors other than psychosis, which might be associated with financial problems.

Our results have to be interpreted in light of some nuances and limitations. Firstly, when referring to “associated factors” or “vicious cycles” we cannot infer purely causal relationships (Burgess et al., 2018). Our use of these terms is rooted in a qualitative context, reflecting participants’ perceptions and experiences. Our analyses allow us to capture depth and variability of personal experience which is often missed in quantitative studies and which highlights potential areas for further investigation. Secondly, participants who approached us to participate in the interviews may have exhibited more pronounced (associations with) financial problems, stronger opinions or better insight into the subject, potentially resulting in a more intense representation of their experiences, compared to non-participants. Thirdly, most participating family members were women and/or parents, which may reflect the prevalent involvement of mothers in the financial challenges of people with psychosis. However, other family members might have different experiences with this topic. Fourthly, we included people with psychosis with wide-varying illness durations. Consequently, participants with longer illness durations may have shared experiences regarding financial problems and their associated factors that are specific to past social and economic conditions (e.g., earlier Dutch social security arrangements). These experiences may therefore be less directly applicable to (other) contemporary societies. Generalizability is, however, not an aim of qualitative research, and the results still provide an insightful and valuable reference point for important stakeholders in other contexts. Lastly, engaging in team-reflexivity regarding our biases (Marguin et al., 2021), and peer debriefing throughout the entire research process, instead of only in a final stage, could enhance insights and foster a more balanced and comprehensive perspective.

To conclude, people with psychosis, family members, and mental healthcare professionals largely mentioned similar, wide-ranging financial problems in people with psychosis. In addition, a number of associated factors were identified. These factors often co-occur and are both general factors and factors specific for psychosis. On one hand, this highlights the increased susceptibility of people with psychosis to financial problems. On the other, financial problems, and their associated factors, amplify each other’s impact, jeopardizing vicious cycles that can negatively affect mental health. These cycles can have serious consequences, including reduced engagement in society, social activities, and treatment. Although financial problems are a common concern within the therapeutic triad, the burden and impact of these problems vary substantially, for example due to different perspectives on the support provided for these problems. This study is an important step towards detection and awareness of financial difficulties and its associated factors among individuals with psychosis. Discussing this topic from multiple perspectives is both complex and crucial, as it helps to bring attention to the issue and fosters a deeper understanding of the challenges faced by this group. Our own tendencies and developments throughout the research process only further emphasize this point. Building on this study, our forthcoming study will shed more light on perspectives of the therapeutic triad on the assistance people with psychosis receive or need for their financial problems. Facilitating collaboration within the therapeutic triad and ensuring the timely recognition of these challenges might contribute to efforts aimed at breaking the vicious cycles of financial problems faced by individuals with psychosis.

## Data statement

Original audio recordings and transcripts will not be made available in order to protect participant confidentiality and privacy. Additional illustrative quotes are available upon request.
